# In Silico Discovery of Antimicrobial Peptides as an Alternative to Control SARS-CoV-2

**DOI:** 10.3390/molecules25235535

**Published:** 2020-11-25

**Authors:** Yamil Liscano, Jose Oñate-Garzón, Iván Darío Ocampo-Ibáñez

**Affiliations:** 1 Research Group of Chemical and Biotechnology, Faculty of Basic Sciences, Universidad Santiago de Cali, Cali 760035, Colombia; jose.onate00@usc.edu.co; 2 Research Group of Microbiology, Industry and Environment, Faculty of Basic Sciences, Universidad Santiago de Cali, Cali 760035, Colombia

**Keywords:** antimicrobial peptides, SARS-CoV-2, spike protein, angiotensin-converting enzyme 2

## Abstract

A serious pandemic has been caused by the severe acute respiratory syndrome coronavirus 2 (SARS-CoV-2). The interaction between spike surface viral protein (Sgp) and the angiotensin-converting enzyme 2 (ACE2) cellular receptor is essential to understand the SARS-CoV-2 infectivity and pathogenicity. Currently, no drugs are available to treat the infection caused by this coronavirus and the use of antimicrobial peptides (AMPs) may be a promising alternative therapeutic strategy to control SARS-CoV-2. In this study, we investigated the in silico interaction of AMPs with viral structural proteins and host cell receptors. We screened the antimicrobial peptide database (APD3) and selected 15 peptides based on their physicochemical and antiviral properties. The interactions of AMPs with Sgp and ACE2 were performed by docking analysis. The results revealed that two amphibian AMPs, caerin 1.6 and caerin 1.10, had the highest affinity for Sgp proteins while interaction with the ACE2 receptor was reduced. The effective AMPs interacted particularly with Arg995 located in the S2 subunits of Sgp, which is key subunit that plays an essential role in viral fusion and entry into the host cell through ACE2. Given these computational findings, new potentially effective AMPs with antiviral properties for SARS-CoV-2 were identified, but they need experimental validation for their therapeutic effectiveness.

## 1. Introduction

Coronaviridae is an enveloped virus family containing positive single-stranded RNA that includes the human coronaviruses (HCoV), identified as causative agents of a wide array of illnesses, including respiratory, enteric, hepatic, and neurological diseases [1,2,3]. Recently, outbreaks of Middle East respiratory syndrome (MERS) and severe acute respiratory syndrome (SARS), caused by Betacoronaviruses (βCoV), MERS-CoV, and SARS-CoV, respectively, emerged and caused outbreaks of severe human respiratory diseases [1,2,3]. In December 2019, a novel HCoV, designated as SARS-CoV-2, was first reported as an atypical pneumonia in Wuhan, China, called COVID-19 [4,5]. Because of its person-to-person transmission and the rapidly increasing number of infected patients worldwide, the World Health Organization (WHO) characterized COVID-19 as a pandemic to promote the implementation of comprehensive strategies for treating and protecting patients [6].

Several clinical trials with agents that demonstrated in vitro preliminary efficacy against SARS-CoV-2 are ongoing [7,8,9,10], but no drugs or vaccines are currently available for treatment and prevention of infection caused specifically by this virus [5,11,12,13,14]. Hence, searching for effective therapeutic treatments for severe illness caused by CoVs, including SARS-CoV-2 [14], is imperative; furthermore, AMPs can be considered promising candidates as potential treatment options. AMPs are a large group of peptides that display remarkable structural diversity and occur naturally to protect hosts against a vast array of microorganisms [15,16,17,18]. AMPs have been identified in several species, from plants to humans, playing a fundamental role in their innate immunity, including antimicrobial activity and immunomodulatory properties [15,16,19,20]. In addition, the fewer side effects, and lower levels of drug tolerance than the other chemical drugs, make AMPs particularly interesting as potential pharmacological compounds for the development of novel therapeutics; currently several AMPs are being evaluated in clinical trials [20,21]. This emerging category of therapeutic agents includes a high diversity of AMPs with antiviral activity, and several studies have evaluated their inhibitory effects on different type of viruses [18,22,23,24]. The mechanisms of action of this AMPs vary widely, including the inhibition of viral entry, and stopping viral fusion through the interactions with structural proteins of the viruses [20,22,25,26]. Indeed, different peptides have been previously evaluated against CoVs, and some of them exhibited specifically anti-SARS-CoV and anti-MERS-CoV in vitro activity, e.g., rhesus and mouse defensins, which mediate their antiviral activity through interaction with the structural viral glycoproteins [3,25,27,28,29,30,31]. Clearly, the AMPs can be excellent candidates as novel treatment options for HCoVs due to their antiviral effects.

In this respect, these viruses have a diversity of genomic elements that encode to structural and non-structural CoV proteins, which are potentially druggable targets to control of this virus family [2,3,25,27]. Four structural proteins compose the CoVs structure, including spike glycoprotein (Sgp), envelope (E) protein, nucleocapsid (N) protein, membrane (M) protein, which play a major role in viral entry, assembly, morphogenesis [2,25]. The Sgp is a glycoprotein located at the viral envelope surface involved in viral binding, fusion, and entry to host cells, while the M protein is also a component of the viral envelope that plays a role in the viral assembly and morphogenesis [2,3,25]. Meanwhile, the E protein is located in the intracellular membranes of the virus and is involved in viral assembly and intracellular trafficking, and the N protein encapsulates the viral genome to form the helical nucleocapsid, located inside the viral envelope [2,3,25]. Despite the fact that the pathogenicity of the CoVs is poorly understood, the host cell receptors recognition mechanisms by viral structural proteins are important determinants of CoVs infectivity, pathogenesis, and host range [2,3,32]. In this respect, initially the Sgp glycoprotein binds to host cell receptors, such as ACE2 for SARS-CoV and SARS-CoV-2 and dipeptidyl peptidase 4 (DPP4) for MERS-CoV, to activate membrane fusion and virus entry [2,3,32,33,34]. After the membrane fusion, the viral RNA is released into the cytoplasm of host cell to allow the replication of the viral genome to produce all the structural envelope proteins, which are necessary to form the assembled virion [2,3,32,35]. Finally, the viral replication cycle is repeated in other cells once the assembled virion are released into extracellular region by exocytosis [3,35].

As a result of the importance of viral structural proteins for the interaction between SARS-CoV-2 and host cells during infection, these proteins can act as therapeutic targets for development of antivirals [2,3,25,32,34]. In this context, the surface structural Sgp glycoprotein is considered an important therapeutic target because of is essential for interaction between the virus and host cell receptor in the viral entry [3,32,33,34,36,37]. The structure of the Sgp protein of SARS-CoV-2 was recently determined. This protein is a trimeric class I fusion protein composed of two subunits: the receptor-binding S1 and membrane fusion S2 [3,32,34]. The S1 and S2 subunits influence viral entry, binding, and fusion. Regarding the mechanism of triggering membrane fusion and virus entry, SARS-CoV-2 requires an initial cleavage at the S1/S2 junction. Then, the receptor-binding domain (RBD) of S1 binds to host cell receptor ACE2, which triggers transition of the S2 subunit to a stable conformation to bring the viral and cell membranes into close proximity and enable fusion [3,32,34]. Hence, interrupting the interaction between the Sgp protein and ACE2 could be important strategies to control virus infection by blocking the viral fusion, cell entry, and viral replication [3]. Recently, several in silico studies have been performed to investigate the interactions between drug compounds and target proteins of SARS-CoV-2 [12,38], but there is no available information on the interactions of AMPs against structural proteins of this virus. However, computational approaches for structural information and protein–peptide interactions of AMPs against MERS-CoV and SARS-CoV, showed significant interactions with the Sgp [27,39,40].

In this study, we investigated the in silico interaction between AMPs and structural glycoproteins of SASR-CoV-2. In particular, we explored the virus-associated protein–peptides docking by focusing on S glyocoprotein of SARS-CoV-2 and a subset of AMPs with particular physicochemical properties. These computational findings are thought to identify new potentially effective molecules with antiviral properties for SARS-CoV-2. Additionally, protein–peptides dockings between the host cell receptor ACE2 and AMPs were performed to evaluate the selectivity of the peptides for viral proteins.

## 2. Results and Discussion

### 2.1. AMPs Clusters, Structural Prediction and Validation

A total of five AMPs clusters, according to their physicochemical characteristics, including net charge, sequence length, percentage of hydrophobicity, and secondary structure, were initially obtained via the K-Means algorithm with a K = 5 (Table 1). All AMPs included in these clusters showed specific physicochemical characteristics and experimental antiviral activity according to APD3 database [18].

Given their physicochemical properties, AMPs included in the cluster four were selected to perform the peptide–protein interaction in order to determine their accuracy in binding with Sgp of SARS-CoV-2, and the host cell receptor ACE2 (Table 1). This cluster included a total of 36 antiviral peptides, which showed three types of secondary structures, including, random coil, α-helix, and beta sheet, net charges that ranged between −3 to 6, and hydrophobicity between 45% and 100% (Table 1).

**Figure 1:**
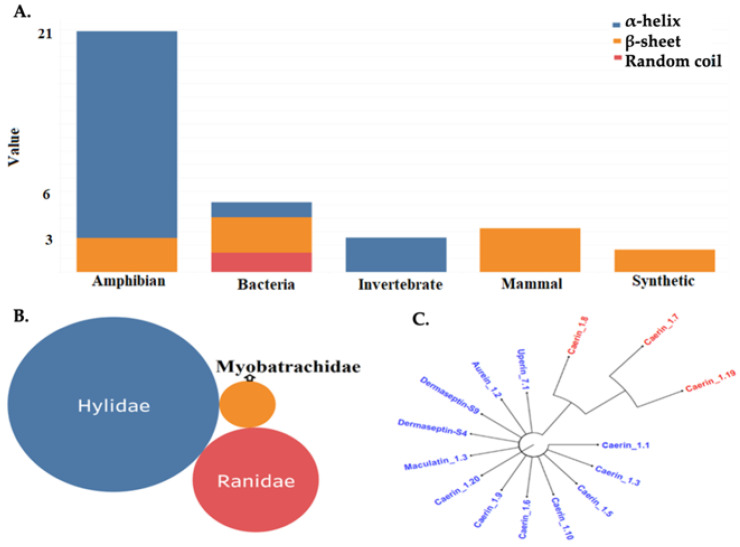
Characteristics from Cluster 4. (**A**) Frequency of organisms and secondary structures in cluster 4; (**B**) Frequency of amphibian families in cluster 4. (**C**) Phylogenetic tree between Hylidae peptides from cluster 4.

Additionally, for these AMPs no experimental hemolytic effect and no anti-SARS-Cov-2 activity have been previously reported [18]. Most of peptides included in this cluster belong to a group of naturally occurring AMPs in amphibians, followed by bacteria and mammal (Figure 1A,B). The phylogenetic analysis showed that the 36 peptides analyzed were clustered into two main clades (Figure 1C), and one of them included 15 peptides that belong to amphibian Hylidae family (Table 2). AMPs are naturally occurring peptides produced as a first line of defense against pathogenic infections by frogs [41,42,43]. Aureins, alyteserins, caerins, citropins, and frenatins are the most abundant AMPs families in frogs of Hylidae family and present high diversity in length and antimicrobial spectras [41,42]. In particular, caerins, aurein, uperin, and maculatin are families of AMPs that have shown in vitro activity against bacteria, virus, fungal and parasites, in addition to anticancer effects [44,45,46,47,48,49]. However, their interaction with SARS-CoV-2 have not been previously evaluated. From these 15 peptides (Table 2), ten belong to caerins family, which are characterized to be α-helix cationic peptides with net charges between +1 and +3, hydrophobicity range 53%–56% and lengths ranging 24–25 residues [44,45,46,47].

The structural models of these 15 AMPs (Table 2) obtained using the I-TASSER platform, were initially validated using RAMPAGE. A total of four AMPs could be validated, including aurein 1.2, caerin 1.3, caerin 1.5, and uperin 7.1, which showed >98% residues outside the favorable region. Remaining 11 AMPs were then optimized using MODELLER to improve their structure (Table 3).

### 2.2. Coordinates for Gridbox of Target Proteins

**Figure 2:**
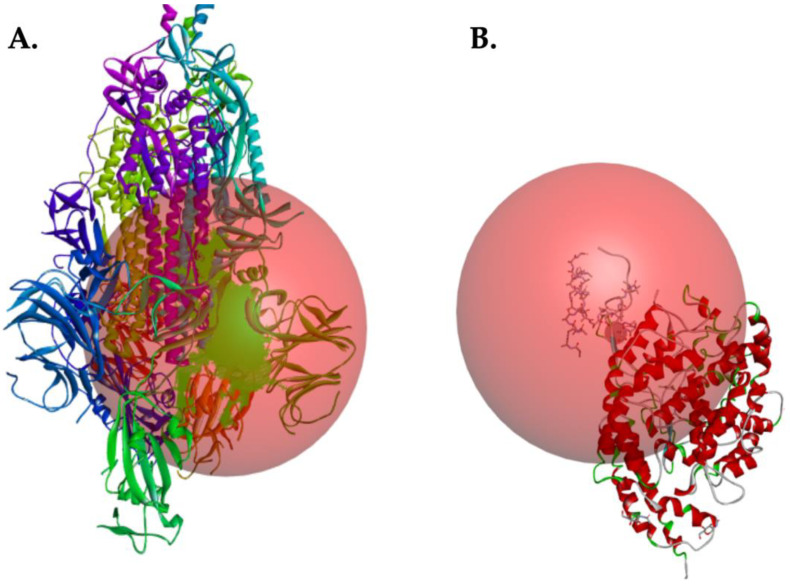
Binding sites of target proteins. Sgp (6VYB) (**A**) and host cell receptor ACE2 (1RL4) (**B**).

The coordinates of the target proteins Sgp protein (6VYB) and host cell receptor ACE2 (1RL4) were obtained with CB-DOCK, as shown in Figure 2 [50]. In this study, we investigated the interactions between AMPs and target proteins SARS-CoV-2 Sgp protein and host cell receptor ACE2. We studied the inhibitory mechanism of a set of AMPs with particular physicochemical characteristics, through peptide-target protein interactions to determine their accuracy in binding with Sgp protein of SARS-CoV-2 and their low affinity for host cell protein ACE2. The binding energies (ΔG) for interactions between each peptide and target proteins are summarized in Table 4 for Sgp and receptor ACE2, respectively. All peptides here evaluated interacted with Sgp (Table 4).

In particular for Sgp, the best interactions were observed for caerin 1.6 and caerin 1.10, with a ΔG of −7.5 kcal/mol and −7.7 kcal/mol respectively (Table 4). For caerin 1.6 the residues VAL17, VAL18, and LYS24 interacted mainly with the residues TYR756, ARG995, and THR998 from viral Sgp. Meanwhile, VAL5, PRO19, GLU23, and LEU25 residues from caerin 1.10 interacted with HIS49, THR51, ASN969, and ARG995 residues of Sgp. The ARG995 was the common residue of Sgp for binding of caerins.

**Figure 3:**
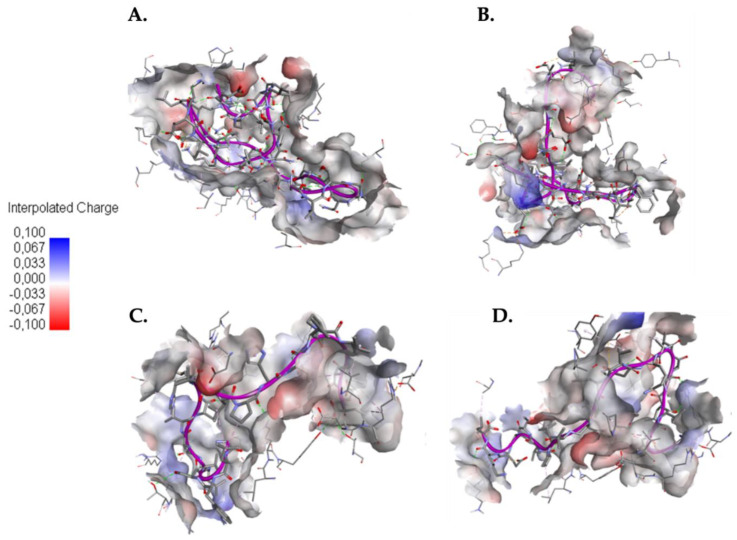
Docking between AMPs and Sgp. (**A**) Interaction between SARS-CoV-HR2P and Sgp; (**B**) Interaction between EK1 and Sgp; (**C**) Interaction between caerin 1.6 and Sgp; (**D**) Interaction between caerin 1.10 and Sgp. Red indicates negative charge and blue indicates positive charge of the Sgp binding site.

In Figure 3 you can see how the SARS-CoV-HR2P control peptides (Figure 3A) and EK1 (Figure 3B) in the binding site present a folding on themselves, which is not observed in the 1.6 (Figure 3C) and 1.10 (Figure 3D) falls. This is because in the control peptides more intramolecular interactions are generated than caerins.

The main type of interaction of the peptides presented in Table 5 was the formation of hydrogen bridges with Sgp, followed by hydrophobic bonds and finally electrostatic interactions. Table 5 shows a low binding affinity between control peptides and Sgp. On the contrary, caerin 1.6 and 1.10 present better affinity with Sgp, among these caerin 1.10 stands out for with a binding energy of −7.7 kcal/mol.

**Figure 4:**
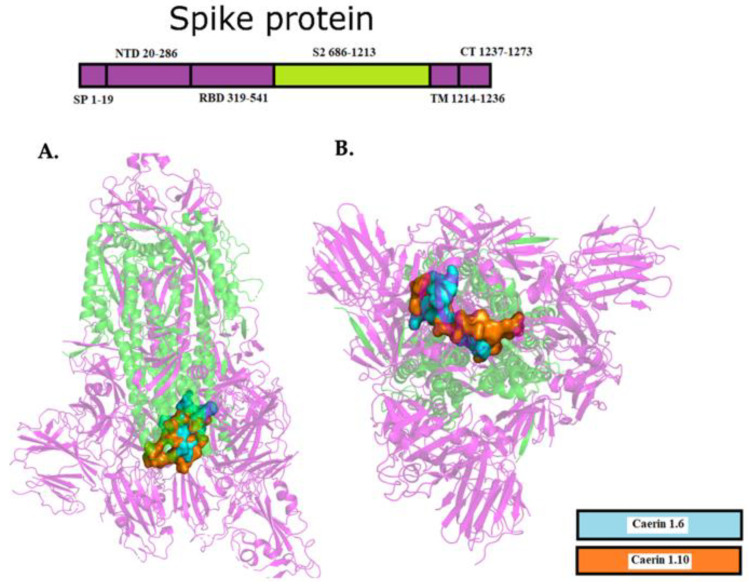
Docking between caerin 1.6 (blue) and caerin 1.10 (orange), and S2 domain (green) of Sgp. (**A**) lateral view of interaction; (**B**) bottom-up view of interaction.

In particular, VAL17 and VAL18 of caerin 1.6, and VAL5 and GLY7 of caerin 1.10 had significant binding with ARG995 in A, B and C chains of Sgp through hydrogen bonds (Figure 4). These peptides blocked in particular the S2 subunit, which together with S1 subunit play an essential role in viral fusion, binding and entry into the cell host due to the cleavage of furin proteases [32,51,52]. In fact, the S1/S2 cleavage site contains several arginine residues which indicates high cleavability [53]. These caerins had a low affinity with ACE2 of −5.4 kcal/mol and −5.2 kcal/mol respectively. Regarding the cell host receptor ACE2, Maculatin 1.3 and Uperin 7.1 showed the best interactions with this target protein, with ΔG of −6.4 and −7.1 kcal/mol, respectively (Table 4).

From the interaction of arginine residues from the Sgp with residues of caerin 1.6 and caerin 1.10, a probable relationship could be inferred between the blocking of interaction between the Sgp and cell host receptor ACE2 by AMPs, and the controlling of viral infection by interrupting the viral fusion, cell entry, and viral replication into human cells [3,32,34,52].

**Figure 5:**
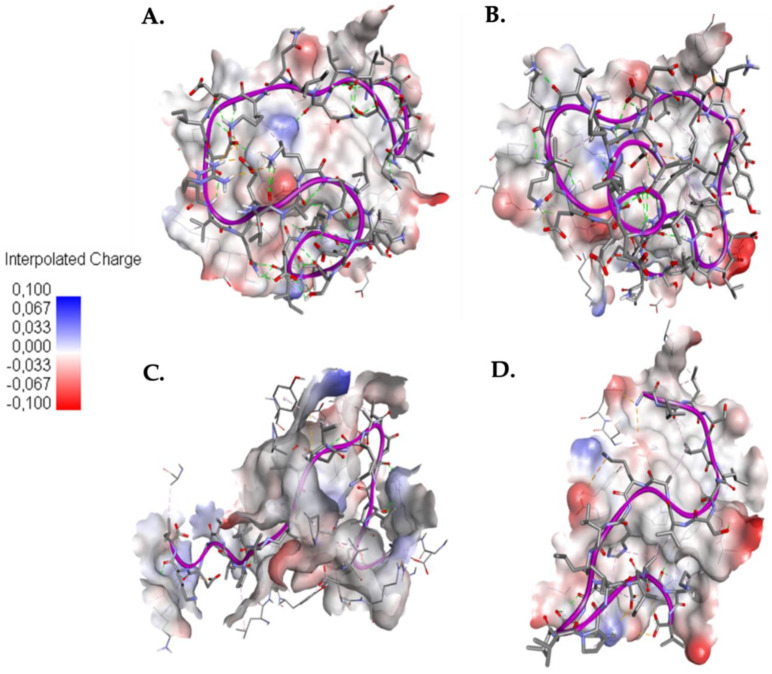
Docking between AMPs and the cell host receptor ACE2. (**A**) Interaction between SARS-CoV-HR2P and ACE2; (**B**) Interaction between EK1 and ACE2; (**C**) Interaction between caerin 1.6 and ACE2; (**D**) Interaction between caerin 1.10 and ACE2. Red indicates negative charge and blue indicates positive charge of the ACE2 binding site.

Regarding the interaction of control peptides and caerins with ACE2 protein, Figure 5 presents a folding over itself of the control peptides (Figure 5A,B), this may be attributed to the formation of more intramolecular interactions with respect to caerins. Also, the presence of negatively charged amino acids in the binding site could cause the formation of intramolecular interactions since the control peptides present a net negative charge.

Table 6 compares the binding energy and interactions between peptides with the ACE2 protein. Increased formation of hydrogen bridges is observed, followed by hydrophobic bonds and electrostatic interactions. With respect to the previous, the caerin 1.10 presented increased formation of hydrophobic bonds than hydrogen bonds. The main ACE2 protein residues that interact with the peptides are ARG482 (forming saline bridges with the glutamic acid or glutamine residues of the peptides), ASP494, TRP163, LYS174, and TYR613.

Given the COVID-19 pandemic, previous studies have shown the in silico and in vitro effectiveness of existing antiviral drugs against SARS-CoV-2, including chloroquine, remdesivir, ivermectins and even antiretrovirals for HIV therapy such as saquinavir [7,8,12,38,54,55,56]. However, no previous studies have reported the interaction of AMPs with SARS-CoV-2 target proteins. In this respect, some studies have previously evaluated the activity of natural and synthetic peptides, including defensins, plectasins, temporins and cathelicidins, against multiple respiratory viruses, such as influenza A virus H5N1, H1N1, MERS-CoV, and SARS-CoV [27,28,29,39,40,57]. Similar to this study, in silico analyses showed the potent antiviral effects of AMPs against Betacoronavirus [27,39]. According to our results, the AMPs are attractive candidates as alternative to conventional antiviral drugs to control SARS-CoV-2 infection, because they offer several potential advantages, including specific anti-CoV effects, high selectivity, and do not be associated with severe adverse effects according to in vitro and in vivo assays [58,59,60].

### 2.3. Interaction between EK1 and SARS-CoV-HR2P and Target Viral Protein

Two peptides, EK1 and SARS-HR2P fusion peptide, with experimentally proven activity against SARS-CoV-2 [61,62,63], were used as control to evaluate and compare the interaction of AMPs (caerin 1.6 and caerin 1.10) with Sgp. Both EK1 and the SARS-CoV-HR2P binding to the HR1 domain present in the Sgp S2 subunit [61,62,63]. Table 6 summarizes the comparison of binding energies between both control peptides and Sgp from SARS-CoV-2. The SARS-CoV-HR2P peptide has a binding energy of −5.5 kcal/mol and the EK1 peptide was −5.3 kcal/mol. The negative net charge of the control peptides summarized in Table 7, appears to be present in their glutamic and aspartic residues, these peptides are the ones that interact more with the residues of the pocket located in the S2 subunit, for example, the GLU21 and GLU28 in the case of SARS-CoV-HR2P peptide and the residues GLU15 and GLU35 in the EK1 peptide. Nevertheless, the high presence of these residues in these peptides did not have the best binding energy when compared with the results presented in the docking of the 1.6 and 1.10 caerin with values of −7.5 kcal/mol and −7.7 kcal/mol respectively, this could be attributed to the fact that the pocket has a greater presence of negatively charged residues such as ASP428 and ASP994, allowing residues such as lysine and histidine from the caerins to achieve better results.

Hydrophobic interactions were more common in the caerins compared to the control peptides. The electrostatic interactions marked the difference between caerin 1.6 and 1.10, with the latter with three more interactions which could have marked the difference shown by the binding energies to Sgp. It is also observed a similarity of interactions between the caerins with the control peptide EK1, for example, the ARG995, ASP994, and the ARG44 are residues of the Sgp that present electrostatic interactions with these peptides. The ARG995 also plays an important role not only in the electrostatic interactions of Sgp with these peptides but also participates in the formation of hydrophobic interactions and hydrogen bonds. Threonines of Sgp are frequently involved in the formation of hydrogen bonds, most frequently THR51 and THR998. Alternatively, in the hydrophobic interactions VAL991 is frequently found interacting with residues of the caerins and EK1 peptide.

### 2.4. Understanding the Role of SER4 and SER8 in the Caerin 1.10

In Table 5 we noticed how almost all the residues of the caerin 1.10 interact with Sgp in the subunit S2 except for S4 and S8, therefore, we decided to modify these serine residues by positive polar residues such as arginine, lysine, and histidine, this because in the pocket of the docking we observed negatively charged residues, all the previous to understand the role of these residues in the peptide. In this way we obtained the synthetic peptide A with ARG4 and ARG8 residues, the synthetic peptide B with H4 and H8 residues, the synthetic peptide C with K4 and K8 residues (Table 7). These residues modified the net charge of the original caerin by increasing it, considering this we modified the original serines of the caerin 1.10 by glycines in such a way that the net charge was not changed in a new synthetic peptide D.

The results of the docking of these synthetic peptides with the Sgp subunit S2 are shown in Table 8, showing that the most notorious change was SR4 and SR8 obtaining a binding energy of −5.0 kcal/mol with the synthetic peptide A that compared to the other synthetic peptides tends to form less hydrogen bridges with Sgp but increases the intramolecular interaction, giving 32 interactions of this type which surpasses the two presented by caerin 1.10. These interactions occur mainly between ARG4 with GLY1 and ARG8 with VAL20, GLU23, GLY7, and ALA22.

**Figure 6:**
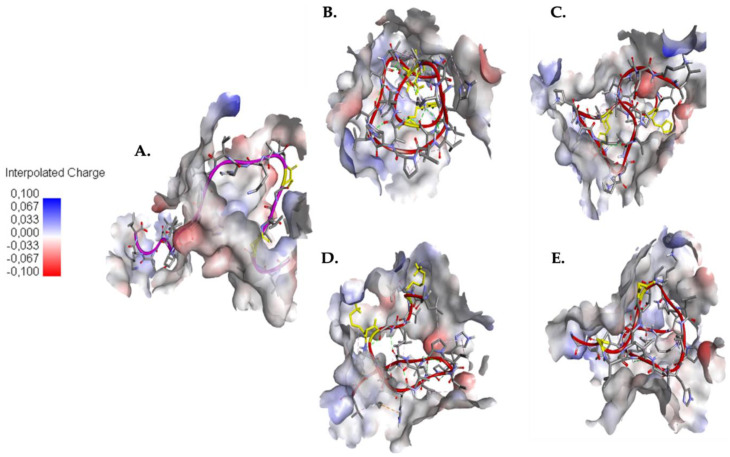
Comparison of the structure and intramolecular interactions between caerin 1.10 and modified caerins. (**A**) caerin 1.10, SER4 and SER8 are highlighted in yellow color; (**B**) caerin 1.10 modified A (SR3-SR8), ARG4 and ARG8 are highlighted in yellow color; (**C**) caerin 1.10 modified B (SH3-SH8), HIS4 and HIS8 are highlighted in yellow color; (**D**) caerin 1.10 modified C (SK3-SK8), LYS4 and LYS8 are highlighted in yellow color; (**E**) caerin 1.10 modified D (SG3-SG8), GLY4 and GLY8 are highlighted in yellow color.

The above is seen more clearly in Figure 6A where the caerin 1.10 is deployed in the pocket while Figure 6B shows us how the synthetic peptide A is compacted by intramolecular interactions. Similarly, the synthetic peptides B, C, and D present a greater number of intramolecular interactions than caerin 1.10 with 9, 23, and 16 interactions respectively. In Figure 6 it is shown how these peptides roll up on themselves diminishing the interaction with the pocket residues, showing that the serine residues S4 and S8 of the caerin 1.10 present a smaller intramolecular interaction which favors a smaller binding energy.

## 3. Materials and Methods

### 3.1. Public Datasets

The computational approach performed in this study involved database screening of AMPs from APD3 antimicrobial database for retrieving their amino acid sequence [18]. Additionally, the crystallographic coordinates for structure of the SARS-CoV-2 S Sgp in the prefusion conformation, and the host cell receptor ACE2 were retrieved from the protein structure database RCSB Protein Data Bank, with PDB ID 6VYB [52] and 1R4L [64], respectively.

### 3.2. Database Screening and Selection of Antimicrobial Peptides

The set of AMPs here investigated were retrieved from the APD3 database. This database contains a total of 3178 AMPs from six kingdoms, including bacteria, archaea, protists, fungi, plants, and animals [18]. According to their in vitro antibacterial, antiparasitic, antiviral, and antifungal activity, a total of 800 AMPs were selected from this database. Predicting the molecular bond between the ligand and the target allows a very efficient virtual examination of the key points of the interaction [43,44]. Deep learning techniques are being used more frequently, which have established a new era of large-scale virtual projection with high efficiency and reliability in in-silico drug design [65]. In in silico molecular binding prediction studies using deep neuronal learning, the multitasking approach is more reliable and the use of target sets with high similarity is preferred [43,44]. Moreover, high similarity sequences also allow the identification of key residues in the ligand–receptor interaction, giving the possibility of applying mutagenesis and improving in this case the affinity of the ligand [66,67].

From this set, we selected a list of AMPs according to their physicochemical properties, such as net charge, percentage of hydrophobicity, length, and secondary structure [27,68,69], using a clustering strategy by integration of the K-Means method and algorithm elbow test with R-Project software Version 1.1.463 [70,71]. When the AMPs did not have available information for their secondary structures, these were predicted using the MLRC method of NPS@: network protein sequence analysis (https://npsa-prabi.ibcp.fr/cgi-bin/secpred_mlr.pl) [72,73]. We selected a cluster according to these criteria: experimental antiviral activity but unknown anti-SARS-CoV-2 activity, non-toxic to mammalian cells, and non-hemolytic effects. Finally, for a subset of 15 peptides from the cluster, a phylogenetic tree was constructed based on AMPs sequences by Maximum likelihood using the MEGA X software, and its reliability was evaluated by bootstrap with 1000 replicates.

### 3.3. In Silico Structural Modeling of AMPs and Validation

First, structural models of the AMPs were obtained using the I-TASSER platform [74]. Here, the 3D atomic models of the peptides were obtained using multiple threading alignments against the protein structure database RCSB PDB [74]. Models with higher confidence according to their C-score was selected [74]. From these structural predictions, a total of 100 molecular models were built for each peptide with MODELLER 9.14 using default parameters. Based on the discrete optimized protein energy score (DOPE score), the best probable structures were selected [75]. Additionally, the stereochemical quality of the best models was verified using Ramachandran plots in PROSA web server [76] and RAMPAGE [77]. All selected models had more than 90% amino acid residues in the favored and additional regions allowed. All the structures analyzed in this study were visualized with PyMOL (https://pymol.org/2/).

### 3.4. AMPs-Target Proteins Docking

The binding modes of AMPs with Sgp, and the host cell receptor ACE2 were determined. To this end, the proteins preparation, the peptides preparation, the grid generation, and the peptide–protein docking were performed using Autodock vina software [78]. For the protein preparation, the target proteins were initially pre-processed by removal of water molecules, addition of Kollman charges, optimization of the Hydrogen bond (H-bond), and addition of Gasteiger charges. The coordinates of grid were obtained by CB-DOCK online tool using the prepared ligand and protein. CB-DOCK is a protein-ligand docking method that identifies the binding sites, calculates the center and size, and customizes the docking box size according to the query ligands [50]. The results obtained were analyzed manually by Discovery Studio Visualizer version 2020 [79]. Two peptides with reported activity against SARS-CoV-2, SARS-CoV-HR2P, and EK1 were used as positive controls, both of synthetic origin and targeting Sgp [61,62,63]. Their 3D structures were created with I-TASSER.

## 4. Conclusions

In conclusion, the results of this study demonstrated that two AMPs (caerin 1.6 and caerin 1.10) have a very high potential to interact with Sgp, but low affinity for ACE2 protein, which suggested the selectivity of these peptides for viral proteins. These AMPs may potentially block the interaction between SARS-CoV-2 S and cell host receptor ACE2, during viral binding, fusion, and entry to host cells, but they need experimental validation for their therapeutic effectiveness.

## Figures and Tables

**Table 1:** Clusters of AMPs found from APD3 Peptide Database.

Cluster	Length		Net Charge		% Hydrophobicity		Total Peptides	Peptides with Antiviral Activity
	Low	High	Low	High	Low	High		
1	17	38	−2	13	35	56	293	48
2	5	38	−7	13	0	40	88	6
3	33	64	−12	20	20	58	178	12
4	6	28	−3	6	45	100	218	36
5	69	147	−11	33	19	49	23	5

**Table 2:** AMPs from Hylidae amphibian Hylidae family in cluster 4.

Peptide	Specie	Structure	Length	Net Charge	% Hydrophobicity	Cluster
Aurein 1.2	*Litoria raniformis*	α-helix	13	1	53	4
Caerin 1.1	*Litoria splendida*	α-helix	25	1	56	4
Caerin 1.3	*Litoria caerula*	α-helix	25	0	56	4
Caerin 1.5	*Litoria caerula*	α-helix	25	1	56	4
Caerin 1.6	*Litoria xanthomera*	α-helix	24	2	58	4
Caerin 1.7	*Litoria xanthomera*	α-helix	24	3	54	4
Caerin 1.8	*Litoria chloris*	α-helix	24	3	54	4
Caerin 1.9	*Litoria chloris*	α-helix	24	2	54	4
Caerin 1.10	*Litoria splendida*	α-helix	25	2	56	4
Dermaseptin-S4	*Phyllomedusa sauvagii*	α-helix	28	4	71	4
Uperin 7.1	*Litoria ewingi*	β-sheet	13	1	61	4
Caerin 1.20	*Litoria caerulea*	α-helix	25	1	56	4
Caerin 1.19	*Litoria gracilenta*	α-helix	25	3	56	4
Dermaseptin-S9	*Phyllomedusa sauvagei*	α-helix	24	4	54	4
Maculatin 1.3	*Litoria eucnemis*	α-helix	21	1	57	4

**Table 3:** Peptide modeling and validation structure. NA: not applicable.

Peptide	Sequence	Itasser	Modeller		Z-Score
		Number of Residues in Favored Region (%)	DOPE Score	Number of Residues in Favored Region (%)	
Aurein 1.2	GLFDIIKKIAESF	100	NA	NA	−0.91
Caerin 1.1	GLLSVLGSVAKHVLPHVVPVIAEHL	78,3	−1399	95	1.09
Caerin 1.10	GLLSVLGSVAKHVLPHVVPVIAEKL	78	−1405	96	0.97
Caerin 1.19	GLFKVLGSVAKHLLPHVAPIIAEKL	91	−1517	100	0.05
Caerin 1.20	GLFGILGSVAKHVLPHVIPVVAEHL	56,5	−1324	96	1.59
Caerin 1.3	GLLSVLGSVAQHVLPHVVPVIAEHL	100	NA	NA	1.27
Caerin 1.5	GLLSVLGSVVKHVIPHVVPVIAEHL	100	NA	NA	1.37
Caerin 1.6	GLFSVLGAVAKHVLPHVVPVIAEK	91	−1422	96	−0.04
Caerin 1.7	GLFKVLGSVAKHLLPHVAPVIAEK	77	−1405	100	−0.18
Caerin 1.8	GLFKVLGSVAKHLLPHVVPVIAEK	96	−1313	96	−1.02
Caerin 1.9	GLFGVLGSIAKHVLPHVVPVIAEK	68	−1577	100	1.34
Dermaseptin-S4	ALWMTLLKKVLKAAAKAALNAVLVGANA	65	−1892	96	−1.73
Dermaseptin-S9	GLRSKIWLWVLLMIWQESNKFKKM	86	−1586	86	0.57
Maculatin 1.3	GLLGLLGSVVSHVVPAIVGHF	89	−1046	100	1.18
Uperin 7.1	GWFDVVKHIASAV	100	NA	NA	1.02

**Table 4:** Interactions between AMPs and SARS-CoV-2 S Sgp. In green highest results. NA: Not applicable.

Sgp		ACE 2	
Peptide	Binding Energies (ΔG) k.cal/mol	Peptide	Binding Energies (ΔG) k.cal/mol
Caerin 1.10	−7.7	Uperin 7.1	−7.1
Caerin 1.6	−7.5	Maculatin 1.3	−6.4
Caerin 1.9	−7.4	Aurein 1.2	−5.9
Uperin 7.1	−7.4	Caerin 1.20	−5.8
Caerin 1.20	−6.9	Caerin 1.3	−5.7
Maculatin 1.3	−6.9	Caerin 1.1	−5.6
Caerin 1.1	−6.7	Caerin 1.5	−5.5
Caerin 1.3	−6.5	Dermaseptin−S4	−5.5
Dermaseptin-S4	−6.4	Caerin 1.6	−5.4
Dermaseptin-S9	−6.4	Caerin 1.9	−5.4
Caerin 1.19	−6.2	Caerin 1.10	−5.2
Caerin 1.5	−6.1	Caerin 1.19	−4.8
Aurein 1.2	−5.8	Dermaseptin-S9	−4.2
Caerin 1.8	−6.0	Caerin 1.7	−6.2
Caerin 1.7	−6.3	Caerin 1.8	−6.2

**Table 5:** Comparison of binding energy and interactions between control peptides and caerins to the Sgp.

Peptides	Binding Energy (kcal/mol)	Hydrogen Bond *	Electrostatic Bond *	Hydrophobic Bond *
SARS-CoV-HR2P	−5.5	B:SER50—ligand:ASN11; B:THR51—ligand:LYS24; B:TYR200—ligand:GLU21; B:TYR200—ligand:ASP17; C:THR739—ligand:GLY4; C:GLN755—ligand:GLU28; C:GLN755—ligand:ASP32; C:GLN755—ligand:ASP32; C:THR761—ligand:ILE5; C:THR761—ligand:ALA7; C:ASN764—ligand:ILE5; B:SER50—ligand:ASN11; A:LEU517—ligand:LYS14; A:GLU516—ligand:AR8; A:THR430—ligand:AR8; B:THR51—ligand:ASN25; B:GLN52—ligand:ASN27; A:ASP428—ligand:LEU36; B:HIS49:C—ligand:VAL10; C:GLY757—ligand:SER8; C:SER758—ligand:VAL9; C:ASN751—ligand:GLU28	A:ARG567—ligand:GLU15; B:LYS202—ligand:ASP17; B:ASP979—ligand:LYS14; A:GLU516—ligand:LYS24	A:PRO426—ligand:LEU36; B:CYS291—ligand:ILE2; B:LYS964—ligand:VAL10; C:LEU752—ligand:ILE31; B:LEU54—ligand:VAL22; A:TYR396—ligand:LEU19; B:TYR200—ligand:LEU19
EK1	−5.3	A:THR430—ligand:SER1; A:LYS462—ligand:GLN4; A:TYR756—ligand:GLU35; A:ARG995—ligand:LEU33; B:THR51—ligand:GLU15; B:GLN52—ligand:TYR14; B:SER975—ligand:LYS17; C:SER750—ligand:THR8; C:TYR756—ligand:LEU36; C:THR761—ligand:ASP11; C:THR998—ligand:GLU35; A:SER514—ligand:SER1; A:THR81—ligand:ASN6; C:THR739—ligand:LEU10; B:ILE973—ligand:LYS25; B:TYR756—ligand:LYS34; C:GLU990—ligand:SER29; A:PHE970—ligand:LYS34; B:HIS49—ligand:GLU15	A:ARG567—ligand:GLU20; A:ARG995—ligand:ASP32; B:ARG44—ligand:GLU15; B:ARG44—ligand:GLU20; B:LYS202—ligand:GLU21; A:ASP428—ligand:SER1; B:ASP994—ligand:LYS34;	A:ARG995—ligand:LYS34; C:VAL991—ligand:ILE31; C:VAL991—ligand:LEU36; C:LEU754—ligand:TYR14;
Caerin 1.6	−7.5	B:THR51—Ligand:SER4; B:ARG44—Ligand:GLY7; B:HIS49 —Ligand:LEU6; B:SER975—Ligand:ALA10; B:ARG983—Ligand:LYS11; B:ARG995—Ligand:VAL17; B:ARG995—Ligand:VAL18; B:THR998—Ligand:ALA22; C:GLN755—Ligand:PRO15; C:TYR756—Ligand:LYS24; A:ASP568—Ligand:GLY7; C:THR998—Ligand:LYS24; A:THR998—Ligand:LYS24; A:ASP428—Ligand:HIS16; B:HIS49—Ligand:VAL5; B:HIS49—Ligand:GLY7; C:GLN755—Ligand:GLY1	B:ASP979—Ligand:LYS11; A:ASP994—Ligand:LYS24	B:ILE973—Ligand:HIS16; C:LEU754—Ligand:PHE3; C:VAL991—Ligand:VAL17; B:VAL991—Ligand:PRO19; C:VAL991—Ligand:VAL20; B:LYS964—Ligand:LEU6; C:ARG995—Ligand:ILE21; C:ARG995—Ligand:LYS24; A:LEU518—Ligand:HIS12; B:HIS49—Ligand:ALA8
Caerin 1.10	−7.7	A:ARG995—ligand:VAL5; A:ARG995—ligand:GLY7; B:HIS49—ligand:GLU23; B:THR51—ligand:LYS24; B:ASN969—ligand:VAL18; B:ASN969—ligand:PRO19; B:THR998—ligand:LEU2; C:GLN755—ligand:HIS16; A:THR998—ligand:LEU2; B:HIS49—ligand:ALA22; B:HIS49—ligand:GLU23; B:VAL991—ligand:LEU6; A:ASP994—ligand:GLY1; B:THR51—ligand:LYS24; B:GLN52—ligand:LYS24;	B:ARG44—ligand:LEU25; B:HIS49—ligand:GLU23; A:ASP994—ligand:GLY1; B:GLU988—ligand:HIS12; C:ASP994—ligand:HIS16;	A:PRO412—ligand:LEU14; B:PRO987—ligand:VAL9; C:PRO987—ligand:PRO15; C:ARG995—ligand:LEU2; B:PRO987—ligand:ALA10; C:LEU754—ligand:PRO19; A:TYR380—ligand:VAL13; C:TYR756—ligand:LEU3; C:VAL991—ligand:HIS16;

* A, B, and C are the chains of the Sgp proteins.

**Table 6:** Comparison of binding energy and interactions between control peptides and caerins to the ACE 2 protein.

Molecule	Binding Energy (kcal/mol)	Hydrogen Bond *	Electrostatic Bond *	Hydrophobic Bond *
SARS-CoV-HR2P	−4.50	Ligand:GLY4—A:TYR158; Ligand:ASP1—A:SER167; Ligand:VAL10—A:SER170; Ligand:ASN11—A:LYS174; Ligand:ILE31—A:GLN472; Ligand:GLN34—A:ARG482; Ligand:ASP32—A:GLU495; Ligand:ASP17—A:THR496; Ligand:ILE2—A:ASN159; Ligand:ASN20—A:ASP471; Ligand:GLN34—A:MET474; Ligand:ASP1—A:TRP163; Ligand:GLU21—A:ASP494	Ligand:GLU35—A:ARG482	Ligand:LYS14—A:LYS174; Ligand:ILE16—A:PRO178; Ligand:LEU19—A:LYS470; Ligand:LEU30—A:LYS475; Ligand:LEU26—A:PRO492; Ligand:LEU36—A:PRO492; Ligand:LEU36—A:ALA614; Ligand:ILE2—A:TRP163; Ligand:LEU36—A:TYR613
EK1	−4.10	Ligand:GLU13—A:TYR158; Ligand:GLU20—A:SER170; Ligand:SER29—A:LYS174; Ligand:SER1—A:ARG482; Ligand:LYS25—A:THR496; Ligand:LEU2—A:TYR613; Ligand:SER1—A:ASP609; Ligand:ASN6—A:PRO492; Ligand:ASN6—A:GLU166; Ligand:GLU20—A:GLU171; Ligand:LYS25—A:GLU495; Ligand:GLU28—A:ASP494; Ligand:SER29—A:ASP494; Ligand:ASP3—A:LYS475; Ligand:SER1—A:SER611; Ligand:LYS25—A:GLU181	Ligand:ASP3—A:LYS475; Ligand:LYS17—A:GLU166	Ligand:PHE9—A:TYR613; Ligand:LYS18—A:PRO135; Ligand:LYS25—A:ARG177; Ligand:LYS24—A:PRO178; Ligand:LYS25—A:PRO178; Ligand:LEU12—A:VAL491; Ligand:LEU12—A:PRO492; Ligand:LYS17—A:LEU162; Ligand:ALA22—A:LYS174; Ligand:LEU19—A:TRP163
Caerin 1.6	−5.40	Caerin 1.6:HIS12—A:ASP609; Caerin 1.6:SER4—A:TYR158; Caerin 1.6:GLU23—A:TRP163; Caerin 1.6:GLU23—A:SER170; Caerin 1.6:ILE21—A:LYS174; Caerin 1.6:VAL13—A:ARG482; Caerin 1.6:ALA22—A:TYR497; Caerin 1.6:LYS11—A:TYR613; Caerin 1.6:HIS12—A:ASP609; Caerin 1.6:VAL18—A:PRO492; Caerin 1.6:PRO19—A:PRO492; Caerin 1.6:GLU23—A:GLU166; Caerin 1.6:GLU23—A:SER167; Caerin 1.6:ALA8—A:ALA614	Caerin 1.6:GLY1—A:ASP157	Caerin 1.6:LEU2—A:LEU162; Caerin 1.6:ALA8—A:PRO492; Caerin 1.6:LYS11—A:PRO492; Caerin 1.6:PRO19—A:VAL491; Caerin 1.6:PRO19—A:PRO492; Caerin 1.6:PHE3—A:VAL491; Caerin 1.6:VAL5—A:TYR255
Caerin 1.10	−5.20	Caerin 1.10:PRO15—A:GLN472; Caerin 1.10:HIS16—A:GLN472; Caerin 1.10:HIS16—A:LYS475; Caerin 1.10:GLU23—A:ARG482; Caerin 1.10:GLU23—A:TYR613; Caerin 1.10:SER8—A:TYR613; Caerin 1.10:LEU2—A:TRP163	Caerin 1.10:LEU25—A:LYS475; Caerin 1.10:GLY1—A:GLU166; Caerin 1.10:LYS11—A:ASP494;	Caerin 1.10:LEU3—A:PRO135; Caerin 1.10:VAL5—A:VAL491; Caerin 1.10:LYS24—A:ALA614; Caerin 1.10:ALA10—A:VAL491; Caerin 1.10:ALA10—A:PRO492; Caerin 1.10:ALA22—A:PRO492; Caerin 1.10:LEU3—A:TRP163; Caerin 1.10:LYS11—A:TYR497; Caerin 1.10:LYS24—A:TYR613

* A is the chain of the ACE2.

**Table 7:** Comparison of physicochemical parameters between control peptides and caerins. Residue changes in caerin 1.10 are highlighted in red.

Peptide	Sequence	Net Charge	Length	Hydrophobicity (%)	Hydrophobic Moment (µH)
Caerin 1.10	GLLSVLGSVAKHVLPHVVPVIAEKL	1.2	25	53	0.28
Caerin 1.10_Synthetic A (SR4, SR8)	GLLRVLGRVAKHVLPHVVPVIAEKL	3.2	25	53	0.28
Caerin 1.10_Synthetic B (SH4, SH8)	GLLHVLGHVAKHVLPHVVPVIAEKL	1.4	25	52	0.27
Caerin 1.10_Synthetic C (SK4, SK8)	GLLKVLGKVAKHVLPHVVPVIAEKL	3.2	25	46	0.34
Caerin 1.10_Synthetic D (SG4, SG8)	GLLGVLGGVAKHVLPHVVPVIAEKL	1.2	25	53	0.28
EK1 (Positive Control)	SLDQINVTFLDLEYEMKKLEEAIKKLEESYIDLKEL	−5.0	36	63	0.34
SARS-CoV-HR2P (Positive Control)	DISGINASVVNIQKEIDRLNEVAKNLNESLIDLQEL	−4.0	36	53	0.37

**Table 8:** Comparison of binding energy and interactions between modified caerins and Sgp.

Peptides	Binding Energy (kcal/mol)	Hydrogen Bonds *	Electrostatic Bond *	Hydrophobic Bond *
Caerin 1.10_Synthetic A	−5.0	B:GLN52—Ligand:ARG4; B:GLN965—Ligand:LYS2; A:ARG567—Ligand:ILE21; B:HIS49—Ligand:GLU23; B:SER967—Ligand:GLU23; B:ASN969—Ligand:ARG8; C:GLY757—Ligand:LEU25; B:PRO39—Ligand:PRO19	B:HIS49—Ligand:GLU23	C:LEU754—Ligand:LEU2; B:LYS202—Ligand:VAL17; B:LYS41—Ligand:VAL18; B:LYS964—Ligand:LYS24; C:LEU754—Ligand:LEU25; A:LEU518—Ligand:LEU6; A:LEU518—Ligand:VAL18; A:LEU518—Ligand:VAL20; B:TYR200—Ligand:VAL17
Caerin 1.10_Synthetic B	−6.8	C:ASP994—Ligand:HIS4; B:ILE973—Ligand:LEU6; B:ILE973—Ligand:GLY7; A:HIS519—Ligand:LYS11; B:GLN52—Ligand:VAL13; A:ASP428—Ligand:ALA22; A:ASP428—Ligand:GLU23; A:ASP427—Ligand:LYS24; A:ASP428—Ligand:LYS24; B:ARG983—Ligand:LEU6; B:GLN992—Ligand:GLY1; B:ARG995—Ligand:LEU3; B:GLY971—Ligand:HIS4; C:GLN755—Ligand:HIS4; C:LEU754—Ligand:HIS16; B:SER968—Ligand:HIS16; B:ILE973—Ligand:HIS4	B:ASP40—Ligand:HIS12	B:ASP40—Ligand:HIS12; B:ILE973—Ligand:LEU6; B:VAL42—Ligand:ALA10; B:LYS41—Ligand:LYS11; A:LEU517—Ligand:ILE21; A:PRO426—Ligand:ALA22; A:PRO463—Ligand:LYS24; A:PRO412—Ligand:LEU2; A:LEU518 —Ligand:VAL9; A:LEU518—Ligand:ILE21; C:PRO986—Ligand:LEU25; C:LEU752—Ligand:HIS4
Caerin 1.10_Synthetic C	−6.1	B:SER967—Ligand:LYS11; C:LEU754—Ligand:HIS12; A:LEU517—Ligand:VAL5; B:ARG44—Ligand:LEU25; B:ARG44—Ligand:LYS24; B:SER975—Ligand:GLU23; B:VAL976—Ligand:GLU23; C:ASN751—Ligand:VAL17; C:GLN755—Ligand:VAL13; C:GLN755—Ligand:LEU14; A:PHE515—Ligand:VAL5; A:GLU516—Ligand:VAL5; B:HIS49—Ligand:LEU25; A:ASP427—Ligand:HIS16	NA	B:TYR200—Ligand:LEU3; A:ASP427—Ligand:HIS16; C:GLN755—Ligand:HIS12; C:LEU752—Ligand:VAL17; B:VAL42—Ligand:LYS24; A:PRO426—Ligand:LEU2; A:PRO463—Ligand:LEU2; A:LEU518—Ligand:LYS8; A:PRO463—Ligand:HIS16; A:PHE464—Ligand:LYS4
Caerin 1.10_Synthetic D	−6.7	B:CYS291—Ligand:GLY1; B:GLN52—Ligand:GLY4; B:GLN52—Ligand:LEU6; B:GLN52—Ligand:VAL9; B:GLY971—Ligand:HIS16; A:ARG567—Ligand:LYS24; B:ARG44—Ligand:ALA22; B:GLN52—Ligand:GLY4; B:GLN52—Ligand:LEU6; B:THR274—Ligand:LEU3; B:SER967—Ligand:VAL20; B:ASN969—Ligand:PRO15; B:ASN969—Ligand:HIS16; B:SER974—Ligand:LEU25; C:GLN755—Ligand:HIS16; B:CYS301—Ligand:GLY1; A:HIS519—Ligand:HIS12; A:ARG567—Ligand:ALA22; A:ARG567—Ligand:HIS12	B:ASP228—Ligand:LYS11; B:ARG44—Ligand:GLU23; B:HIS49—Ligand:GLU23	C:LEU754—Ligand:VAL5; C:LEU754—Ligand:PRO19; A:LEU518—Ligand:LYS11; A:LEU518—Ligand:VAL13; B:LYS964—Ligand:ILE21

* A, B, and C are the chains of the Sgp proteins. NA: not applicable.
